# Rheumatoid arthritis is associated with increased DKK1 expression and disturbances in the bone turnover regulating genes

**DOI:** 10.1186/1479-5876-9-S2-P53

**Published:** 2011-11-23

**Authors:** Joana Caetano-Lopes, Ana Rodrigues, Ana Lopes, Ana C Vale, Michael A Pitts-Kiefer, Bruno Vidal, Inês P Perpétuo, Jacinto Monteiro, Yrjö T Konttinen, Maria F Vaz, Ara Nazarian, Helena Canhão, João E Fonseca

**Affiliations:** 1 Rheumatology Research Unit, Instituto de Medicina Molecular, Faculdade de Medicina da Universidade de Lisboa, Lisbon, Portugal; 2 Serviço de Reumatologia e Doenças Ósseas Metabólicas, Hospital de Santa Maria, Lisbon, Portugal; 3 Dept. of Mechanical Engineering, Instituto Superior Técnico, ICEMS, Lisbon, Portugal; 4 Center for Advanced Orthopaedic Studies, Dept. of Orthopaedic Surgery, Beth Israel Deaconess Medical Center, Harvard Medical School, Boston, Massachusetts, USA; 5 Orthopaedics Dept., Hospital de Santa Maria, Lisbon, Portugal; 6 University of Helsinki, Dept. of Medicine, ORTON Orthopaedic Hospital of the Invalid Foundation, Helsinki; COXA Hospital for Joint Replacement, Tampere, Finland

## Background

Rheumatoid arthritis (RA) and primary osteoporosis (OP) are associated with bone fragility. In this study we aimed to identify differences in the mechanisms involved in bone fragility by comparing gene expression between RA and OP bone samples.

## Materials and methods

RA patients submitted to hip replacement surgery were recruited. They were matched to a group of primary OP patients for bone mineral density and major clinical fracture risk factors (age, gender, BMI). Trabecular bone microarchitecture was assessed by micro-computed tomography and bone mechanical behavior by compression tests. Bone cell activity was analyzed by studying gene expression.

## Results

Seventeen patients were included, ten with RA and seven with primary established OP. Bone microarchitecture and mechanical bone properties did not differ between groups. RA bone microenvironment, compared to primary OP, had a gene expression profile characterized by upregulated pro-osteoclastogenic cytokines and DKK1, paralleled by raised expression of factors that promote osteoblastic activity.

## Conclusions

Bone fragility in RA patients is induced by an unbalanced bone turnover that is qualitatively different from the pathobiologic phenomena that occur in primary OP. The type of bone gene disturbances is suggestive of a pivotal role for DKK1 in this process, suggesting that it could be used as a therapeutic target to prevent RA bone damage.

